# Supplementary material for: The interactome of intact mitochondria by cross-linking mass spectrometry provides evidence for coexisting respiratory supercomplexes
Source: Mol Cell Proteomics. 2017 Dec 8;17(2):216–32. doi: 10.1074/mcp.RA117.000470 (PMC5795388; doi:10.1074/mcp.RA117.000470)
Supplement: Supplemental Data [file supp_RA117.000470_133922_0_supp_23978_fzffwf.zip › spectra_annotation/mito_DR_spectra_annotation/19-1-4-1-10-1.pdf]

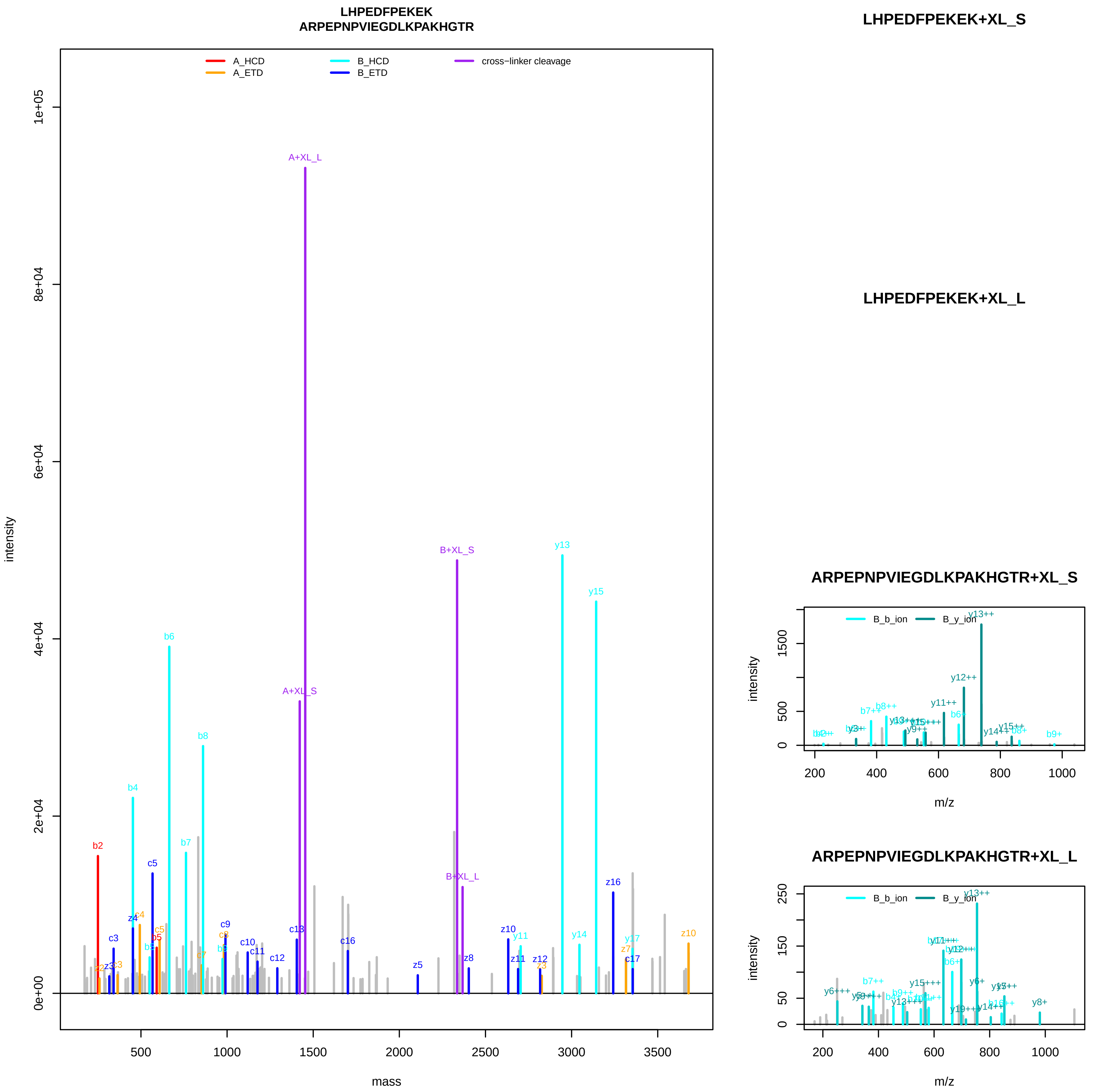
LHPEDFPEKEK
ARPEPNPVIEGDLKPAKHGTR
A_HCD
A_ETD
B_HCD
B_ETD
cross−linker cleavage
intensity
mass
0e+00
2e+04
4e+04
6e+04
8e+04
1e+05
500
1000
1500
2000
2500
3000
3500
A+XL_L
A+XL_S
B+XL_S
B+XL_L
b2
b4
b5
b6
b7
b8
b9
c3
c4
c5
c8
c9
c10
c11
c12
c13
c16
c17
z3
z4
z5
z7
z8
z10
z11
z12
z16
y11
y13
y14
y15
y17
z10
LHPEDFPEKEK+XL_S
LHPEDFPEKEK+XL_L
ARPEPNPVIEGDLKPAKHGTR+XL_S
B_b_ion
B_y_ion
intensity
m/z
0
500
1500
200
400
600
800
1000
y13++
y12++
y11++
b6+
b7++
b8++
b3+
y9++
y14++
y15++
b9+
ARPEPNPVIEGDLKPAKHGTR+XL_L
B_b_ion
B_y_ion
intensity
m/z
0
50
150
250
200
400
600
800
1000
y13++
y6+++
b7++
y15+++
y6+
y8+
